# Enhancing Permanence of Corrosion Inhibitors Within Acrylic Protective Coatings for Outdoor Bronze Using Green Nanocontainers

**DOI:** 10.3390/molecules29235702

**Published:** 2024-12-03

**Authors:** Giulia Pellis, Fabrizio Caldera, Francesco Trotta, Thais Biazioli de Oliveira, Paola Rizzi, Tommaso Poli, Dominique Scalarone

**Affiliations:** Department of Chemistry, University of Torino, Via Pietro Giuria 7, 10125 Torino, Italy; giulia.pellis@unito.it (G.P.); fabrizio.caldera@unito.it (F.C.); francesco.trotta@unito.it (F.T.); thabiazioli@hotmail.com (T.B.d.O.); paola.rizzi@unito.it (P.R.); tommaso.poli@unito.it (T.P.)

**Keywords:** cyclodextrins, corrosion inhibitors, bronze statues, inclusion complexes, protective coatings

## Abstract

Outdoor bronze statues are constantly exposed to weather conditions and reactive compounds in the atmosphere that can interact with their surfaces. To avoid these interactions, a commonly used method is the application of coatings with corrosion inhibitors. However, a significant limitation of these inhibitors is their gradual loss over time. In this study, we aimed to improve the durability of 5-ethyl-1,3,4-thiadiazol-2-amine (AEDTA), the inhibitor chosen to formulate new acrylic coatings for outdoor bronzes. Methyl-β-cyclodextrin (Me-β-CD) was selected to host the inhibitor due to the capability of cyclodextrins to form complexes incorporating small organic molecules. The complexes of Me-β-CD and AEDTA were prepared and the inclusion of AEDTA was proved by Fourier-transform infrared spectroscopy, X-ray diffraction and nuclear magnetic resonance spectroscopy. Then, acrylic coatings were prepared at different concentrations of the Me-β-CD/AEDTA system. They were thermally aged and monitored every 24 h. To evaluate the volatilization of the corrosion inhibitor, solid phase microextraction-gas chromatography/mass spectrometry (SPME-GC/MS) and thermal desorption-GC/MS (TD-GC/MS) analyses were performed during the first 72 h. The results were compared to those of pure AEDTA films and Incralac^®^. The outcomes showed that Me-β-CD/AEDTA complexes are promising candidates for developing coatings with improved stability and longer retention of AEDTA.

## 1. Introduction

Bronze statues are among the most widespread forms of outdoor public art in urban environments. However, their conservation poses significant challenges due to the lack of funding and the limited effectiveness of conservation treatments over time. These artworks are often coated with either natural or artificial patinas, but in the long term this does not fully prevent degradation processes driven by external factors, including pollution, UV rays, and humidity. One of the most common approaches to mitigate these effects involves the application of protective coatings. However, the most widely used acrylic coating for bronze restoration, Incralac^®^, contains benzotriazole (BTA), a compound that is both toxic and suspected of being carcinogenic [1]. Therefore, there is a need for the development of safer, more effective, and long-lasting conservation strategies, which require the replacement of hazardous materials currently in use [2,3].

Among the non-toxic, cost-effective alternatives to BTA, 5-ethyl-1,3,4-thiadiazol-2-amine (AEDTA) has been proposed since it has been shown to reduce the corrosion of copper in 3.0% NaCl solutions [4]. A more recent study has identified AEDTA as a promising substitute of BTA, being safer, more stable, and with a lower tendency to leave the coating over time [5]. In this study, we propose to improve the permanence of AEDTA in acrylic coatings using methyl-β-cyclodextrin (Me-β-CD) as a green nanocontainer for the corrosion inhibitor.

Cyclodextrins (CDs) have gathered significant attention across various scientific disciplines due to their versatility. These water-soluble, macrocyclic oligosaccharides are composed of at least six α-D-glucopyranose units linked via α-(1→4)-glycosidic bonds and are produced through the enzymatic degradation of starch by cyclodextrin glucanotransferase (CGTase) [6]. The outer surface of the molecule is hydrophilic, rendering it water-soluble, while the inner cavity is less hydrophilic, creating a favourable environment for non-polar compounds. Various CD derivatives, such as hydroxypropyl-β-CD and methyl-β-CD, have been developed to improve their performance, including enhanced complexation efficiency and controlled release properties [7]. CDs are extensively used in the pharmaceutical, biomedical, and food industries, and their unique ability to form inclusion complexes with guest molecules has expanded their applications by improving the solubility, stability, and bioavailability of these molecules [8]. Several studies have demonstrated the potential of CDs to host corrosion inhibitors, which are typically small organic molecules [9]. The use of CDs as complexing agents aims to increase the retention of corrosion inhibitors within protective coatings, as these inhibitors often separate and leach out of the film, losing their effectiveness [10]. By incorporating corrosion inhibitors into carriers, such issues can be mitigated [11]. Research has shown that CDs are reliable carriers for this purpose: β-CD has successfully encapsulated 5-mercapto-1-phenyl-tetrazole to improve bronze corrosion resistance [12]; α- and β-CD complexes with dibenzylthiourea (DBT) enhanced carbon steel corrosion protection by improving DBT’s solubility [13]; and β- and γ-CDs complexes with various organic inhibitors have demonstrated improved corrosion resistance for aluminium alloys and zinc [14,15,16,17]. Additionally, β-CD complexes with 2-phosphonobutane-1,2,4-tricarboxylic acid have shown enhanced corrosion protection for carbon steel [18], while hydroxypropyl-β-CD complexes with octadecylamine (ODA) have improved anticorrosion properties for steel due to the increased solubility of the guest [19].

**Figure 1:**
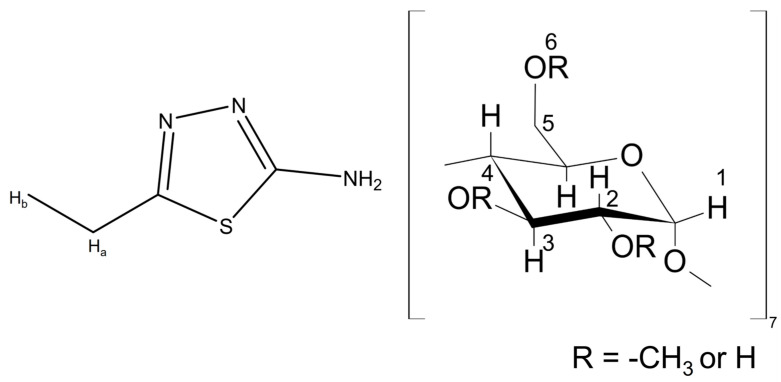
5-ethyl-1,3,4-thiadiazol-2-amine (AEDTA) and methyl-β-cyclodextrin.

Based on these studies, we sought to investigate the potential of methyl-β-cyclodextrin (Me-β-CD) as a green nanocontainer for the AEDTA corrosion inhibitor (Figure 1), with the aim of improving its retention within Paraloid^®^ B44 acrylic coatings. 

Methyl-β-cyclodextrin, due to its methoxy groups in place of primary hydroxyls, offers better compatibility with acrylic resins than β-CD. The complexes of methyl-β-cyclodextrin and AEDTA were successfully synthesized, and the inclusion of AEDTA was confirmed by Fourier-transform infrared spectroscopy (FTIR), X-ray diffraction (XRD), and nuclear magnetic resonance (NMR) spectroscopy. The stoichiometry of the complex was determined using the continuous variation method [19]. Furthermore, three types of acrylic coatings containing varying concentrations of the methyl-β-cyclodextrin/AEDTA complex were prepared and applied to both inert substrates and polished bronze discs. These samples underwent accelerated aging at 80 °C, monitoring chemical changes every 24 h by FTIR in both transmission and reflection modes, depending on the substrate. Solid phase microextraction-gas chromatography/mass spectrometry (SPME-GC/MS) and thermal desorption-GC/MS (TD-GC/MS) were used to assess the volatilization of the corrosion inhibitor from the acrylic matrix, comparing the Me-β-CD/AEDTA complex to AEDTA alone. Both systems were then compared with Incralac^®^.

## 2. Results and Discussion

The research plan envisaged three main phases: (1) the investigation of the interaction between methyl-β-cyclodextrin and AEDTA, (2) the determination of the stoichiometry of the methyl-β-cyclodextrin/AEDTA complex, and (3) the evaluation of the prolonged permanence within the coating of the corrosion inhibitor hosted in the methyl-β-cyclodextrin cavity.

### 2.1. Methyl-β-Cyclodextrin/AEDTA Complex Characterization

The interaction between methyl-β-cyclodextrin and AEDTA was investigated by FTIR, XRD, and NMR spectroscopy, well-established techniques for studying cyclodextrin inclusion complexes [12]. The methyl-β-cyclodextrin/AEDTA complex with a 1:1 molar ratio was extensively analyzed. To further confirm the interaction between the host and guest molecules, a physical mixture with the same molar ratio was also prepared.

**Figure 2:**
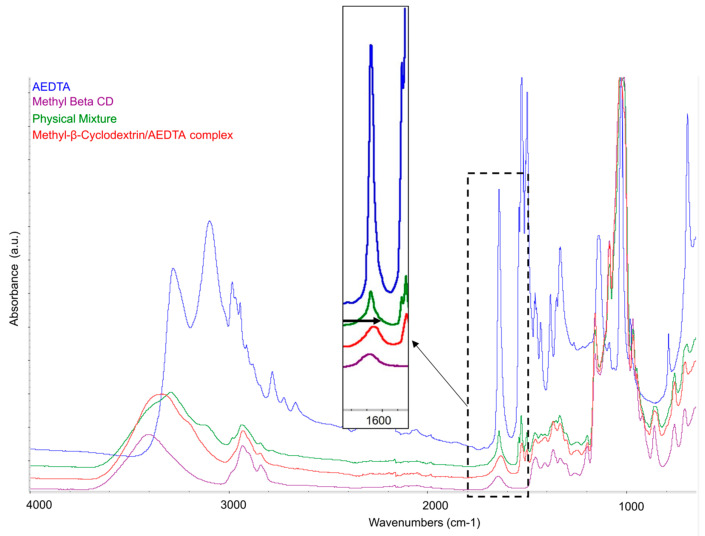
ATR-FTIR spectra of AEDTA, Me-β-CD, their complex, and physical mixture. The box shows the spectral region with the characteristic absorption band of AEDTA and its shift due to complexation.

It has been vastly reported that the variation of the shape, shift, and intensity of the FTIR absorption bands of the guest or host molecule can provide information concerning the formation of complexes [20,21]. Figure 2 illustrates the infrared spectra of methyl-β-cyclodextrin, AEDTA, and of the binary systems (complex and physical mixture). The characteristic AEDTA absorption peak at 1636 cm⁻¹, attributed to the scissoring vibration of the amino group [20,22], shifted and changed in shape in the spectrum of the complex. The broadening of the same peak suggested an amorphization of AEDTA, a reasonable consequence of its complexation, while the shift toward lower wavenumbers reflected a change in the vibration energy of the amino group. These changes in the characteristic absorption band of the corrosion inhibitor highlighted the occurring of an interaction and opened two potential scenarios: the formation of an inclusion or non-inclusion complex. Conversely, the spectrum of the physical mixture perfectly overlapped with the spectra of the individual components, and the peak at 1636 cm^−1^ remained sharp and centred at the same wavenumber of the AEDTA reference, indicating no significant interaction between the two components.

**Figure 3:**
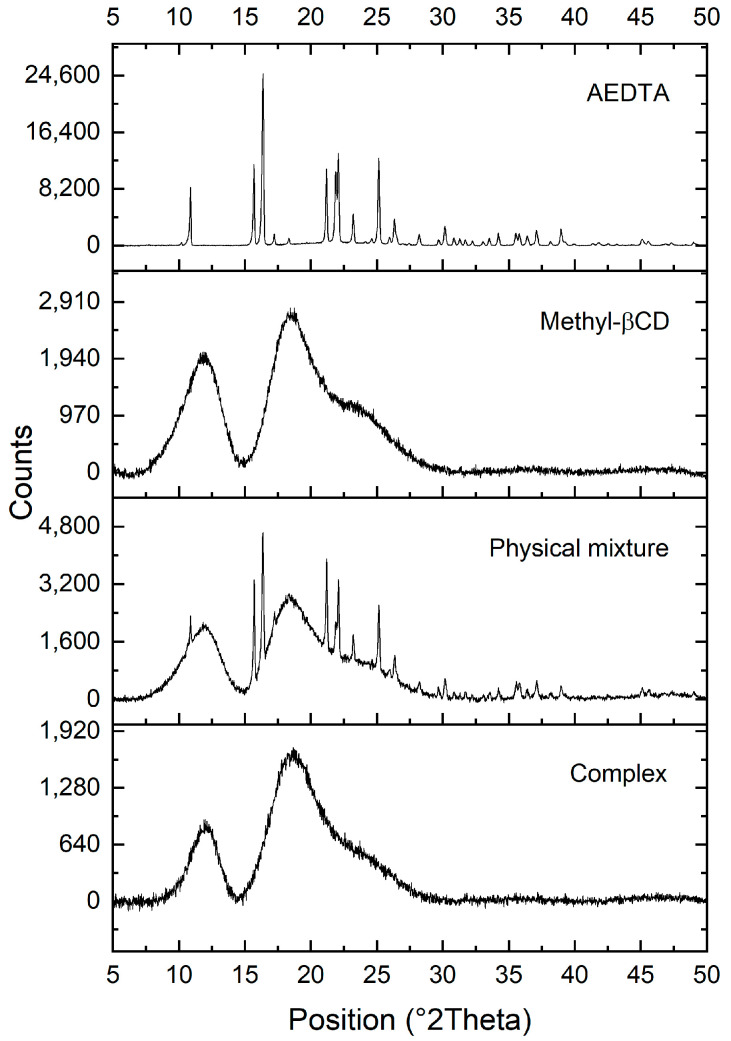
XRD diffractograms of AEDTA, methyl-β-cyclodextrin, their complex, and their physical mixture.

The successful complexation of AEDTA and methyl-β-cyclodextrin was confirmed by XRD, a useful technique for the detection of cyclodextrin complexation in a powder or microcrystalline state [23,24]. The XRD diffractograms are reported in Figure 3: the diffraction pattern of AEDTA exhibited several sharp peaks, indicating its crystalline structure, while the diffractogram of cyclodextrin was characteristic of an amorphous material. As expected, the XRD pattern of the physical mixture was a direct superimposition of those of the individual components, whereas the methyl-β-cyclodextrin/AEDTA complex showed two broad halos in the diffraction similar to those of methyl-β-cyclodextrin, indicating its amorphous state. This loss of crystallinity suggested that AEDTA and methyl-β-cyclodextrin were mixed at a molecular level, providing further evidence of complexation [25].

**Figure 4:**
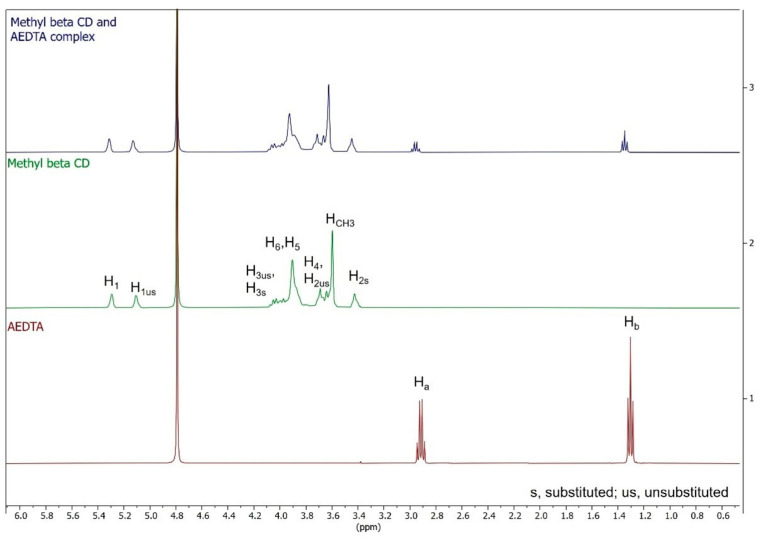
^1^H-NMR spectra (600 MHz, D_2_O) of AEDTA, methyl-β-cyclodextrin, and their complex.

The nature of the interaction between AEDTA and methyl-β-cyclodextrin was also explored using ^1^H NMR spectroscopy, a powerful method for analyzing supramolecular assemblies in solution. Significant chemical shift changes in specific host or guest nuclei between the free and bound states can confirm the formation of inclusion complexes in solution [7]. Figure 4 presents the NMR spectra of AEDTA, methyl-β-cyclodextrin, and the methyl-β-cyclodextrin/AEDTA complex (1:1 molar ratio), with the chemical shifts (δ) and displacements (Δδ) detailed in Table 1 [26].

**Figure 5:**
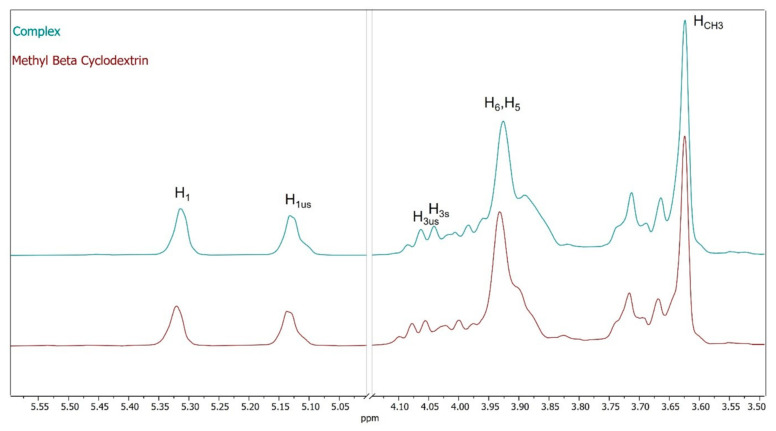
Zoom in on NMR spectra of methyl-β-cyclodextrin and the complex.

The inclusion of AEDTA in the methyl-β-cyclodextrin cavity was indicated by the chemical shift variations in the host protons H_3_ and H_5_, compared to their positions in free CD molecules (Figure 5). Both cyclodextrin protons were shielded, signifying the inclusion of an electron-rich group, likely the -NH₂ group of AEDTA. The signals due to the more external protons, H_1_ and the protons of the methoxy groups (Figure 5), underwent a minor shift, further indicating that the guest interacted with the internal cavity. The NH_2_ signal of AEDTA could not be seen in the NMR spectra because the amino group had exchangeable protons that broadened the signals. Thus, no direct interaction between these protons and the host was detectable by NMR. Nonetheless, the change in the amino group scissoring vibration, observed in the FTIR spectra, supported the hypothesis of an interaction between AEDTA and methyl-β-cyclodextrin involving the NH₂ group and the cyclodextrin internal protons, while the thiadiazole ring and ethyl group of AEDTA remained external to the cavity.

It is worthy of note that no new peaks were observed in the NMR spectrum of the complex, suggesting that the inclusion of AEDTA in methyl-β-cyclodextrin occurred with an exchange process in the NMR timescale [12].

### 2.2. Study of the Methyl-β-Cyclodextrin/AEDTA Complex

To gather information on the stoichiometry of the complex, thirteen complexes were synthetized employing different host/guest molar ratios (Table 2, Section 2.2).

**Figure 6:**
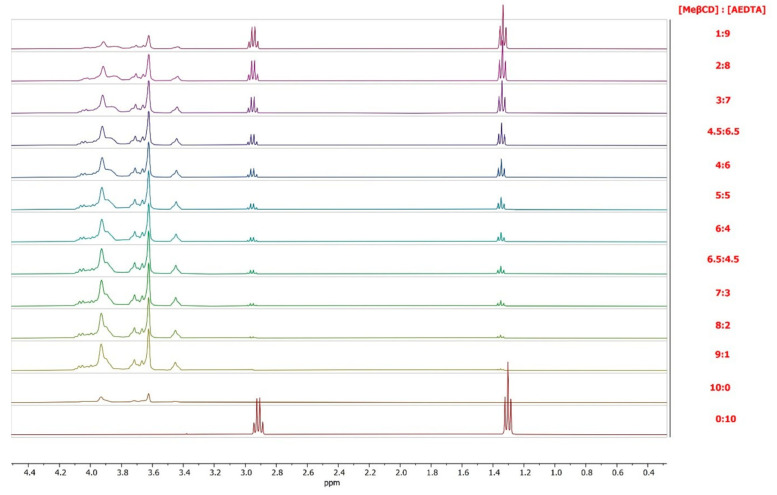
NMR spectra of AEDTA, methyl-β-cyclodextrin, and their complex.

^1^H NMR spectra of pure components and methyl-β-cyclodextrin/AEDTA complexes are reported in Figure 6.

The stoichiometry of the complex was determined using the continuous variation method, originally proposed by Paul Job (1928) [27]: this method is based on induced chemical shift variation, Δδ, that is experimentally determined, and it is directly related to the concentration of the complex. Δδ is defined as the difference between the chemical shifts of the free molecule and the bounded one.

NMR spectra were recorded for a series of solutions in which the total concentration of the two species was kept constant at 3 mM and the concentration ratio of the single components (r = [AEDTA]/([AEDTA] + [Me-β-CD]) or r = [Me-β-CD]/([AEDTA] + [Me-β-CD]) was varied between 0 and 1.

**Figure 7:**
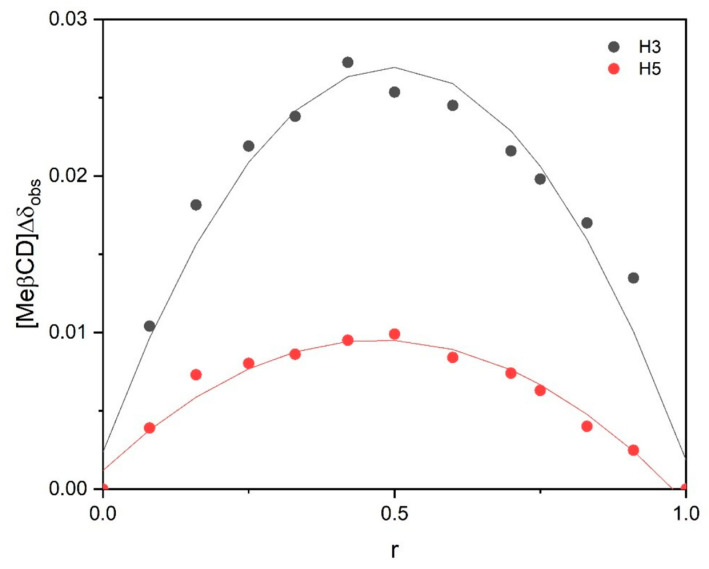
Job’s plot of H_3_ and H_5_ of Me-β-CD.

Under these conditions, if a physical quantity directly related to the concentration of the complex can be measured and plotted as a function of r, its maximum value will be reached at r = m/m + n, where m and n are AEDTA and methyl-β-cyclodextrin proportions in the complex, respectively. When signals are rapidly averaged by the exchange between free and bound states, the quantity Δδ_obs_[AEDTA] or Δδ_obs_[Me-β-CD] (where Δδ_obs_ is the chemical shift difference between free AEDTA or Methyl-β-Cyclodextrin and the observed value for a given ratio r) will be proportional to the complex concentration. Therefore, Δδ_obs_[AEDTA] or Δδ_obs_[Me-β-CD] can be plotted against r [28,29]. Figure 7 highlights the most significantly affected chemical shifts. The continuous variation plot of Δδ_obs_[Me-β-CD] against r of the internal protons H_3_ and H_5_ showsed a maximum at r = 0.5, indicating a 1:1 complex stoichiometry.

A corresponding Job’s plot for the NH₂ protons could not be drawn, as no visible signal was observed in the spectra. However, it is reasonable to assume that the complex followed an equimolar host–guest ratio.

### 2.3. Characterization of Acrylic Coatings Containing Methyl-β-Cyclodextrin/AEDTA Complex

To ascertain whether the complexation of the corrosion inhibitor enhances its permanence within acrylic coatings, coatings made of Paraloid^®^ B44 and containing 5%, 15%, and 20% *w*/*w* of the methyl-β-cyclodextrin /AEDTA complex were prepared from the solutions described in paragraph 2.4. These coatings were then thermally aged at 80 °C, and AEDTA volatilization was monitored using FTIR, SPME-GC/MS, and TD-GC/MS.

**Figure 8:**
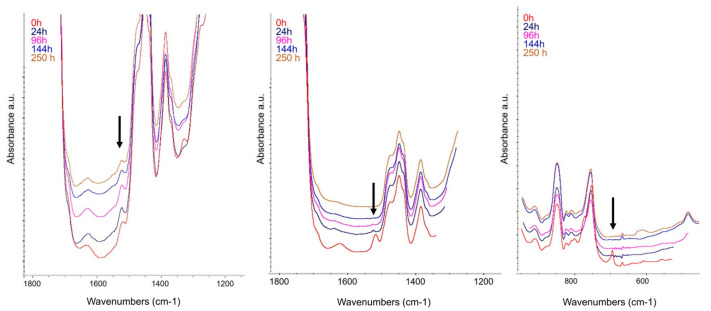
FTIR monitoring during aging at 80 °C: acrylic coating with 2% *w*/*w* of complexed AEDTA (**left**); acrylic coating with 5% *w*/*w* of uncomplexed AEDTA (**centre**), and Incralac^®^ (**right**).

In Figure 8, the FTIR spectra recorded during the first 250 h of aging of the coatings containing 20% *w*/*w* of the complex, which corresponded to 2% *w*/*w* of AEDTA, were compared to the spectra of coatings containing 5% *w*/*w* of uncomplexed AEDTA and to the spectra of Incralac^®^. For clarity, only part of the FTIR spectra is shown.

In the coating with the complex, the characteristic peak of AEDTA at 1636 cm^−1^ remained visible throughout the monitoring period, whereas in the coating with uncomplexed AEDTA, the same peak diminished rapidly and disappeared entirely after 100 h of aging. Similarly, in Incralac^®^, the characteristic BTA peak at 740 cm⁻¹, attributed to the CH stretching vibration of the aromatic ring [30], decreased in intensity shortly after aging began. This demonstrates that including AEDTA in methyl-β-cyclodextrin significantly extends the inhibitor retention within the coating. Further confirmation of the improved coating performance was provided by TD-GC/MS and SPME-GC/MS analyses.

**Figure 9:**
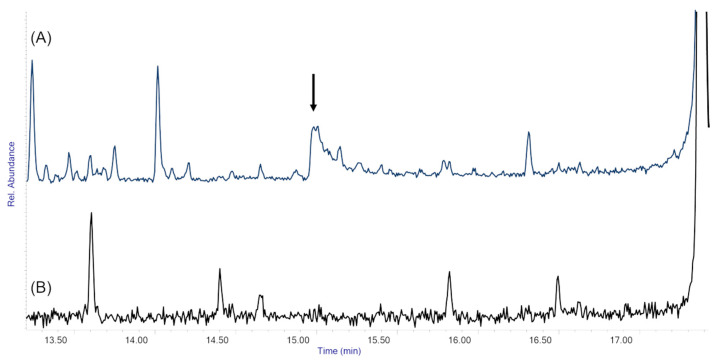
Detail of SPME-GC/MS curves obtained from acrylic coatings treated with uncomplexed AEDTA: (**A**) unaged coating, (**B**) coating aged 72 h at 80 °C. The arrow highlights the AEDTA peak.

SPME-GC/MS results are shown in Figure 9. Acrylic coatings treated with AEDTA released the inhibitor over time: in the headspace analysis of the unaged coating, AEDTA was clearly detected (Figure 9A), while the analysis of the coating thermally aged for 72 h did not show any AEDTA peak, which means that the inhibitor had left the coating (Figure 9B). In contrast, the chromatogram of the coating containing the methyl-β-cyclodextrin/AEDTA complex (data not shown) revealed no AEDTA peaks at either time zero or after 72 h of thermal aging. This result suggests that complexation prolongs the retention of AEDTA within the coating.

**Figure 10:**
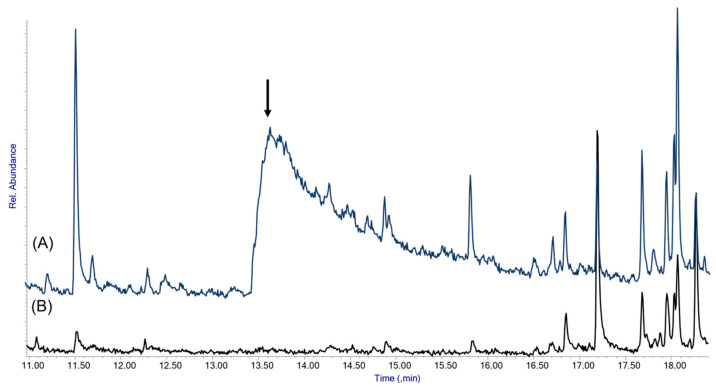
Detail of the TD-GC/MS analysis of acrylic coatings aged 72 h at 80 °C and treated with the methyl-β-cyclodextrin/AEDTA complex (**A**) and with uncomplexed AEDTA (**B**). The arrow highlights the AEDTA peak in the coating containing the inclusion complex.

The presence or absence of the inhibitor in the coatings was double-checked by TD-GC/MS analysis at 250 °C (Figure 10). In coatings with uncomplexed AEDTA aged for 72 h, no AEDTA peak was observed, confirming that the corrosion inhibitor went away. On the contrary, the coating containing the methyl-β-cyclodextrin/AEDTA complex showed the peak of the corrosion inhibitor, providing clear evidence that complexation improves the permanence of the corrosion inhibitor in the coating. Based on these results, the use of cyclodextrins as nanocontainers for corrosion inhibitors offers a highly effective and promising approach for developing long-lasting anti-corrosion coatings suitable for protecting outdoor bronze artworks.

## 3. Materials and Methods

### 3.1. Materials

Paraloid^®^ B44 (ethyl acrylate/methyl methacrylate copolymer) was purchased from Sinopia s.a.s., 5-ethyl-1,3,4-thiadiazol-2-amine (AEDTA) (MW 129.18 g/mol) from Sigma Aldrich (St. Louis, MO, USA), and methyl-β-cyclodextrin with 0.5% of methylation was kindly provided by Roquette Freres (MW 1190 g/mol). The solvent used to prepare the coatings was 1-methoxypropan-2-ol, purchased from Sigma Aldrich.

### 3.2. Synthesis of Methyl-β-Cyclodextrin/AEDTA Complexes

The complexes were synthetized at different molar concentrations (Table 2). At first, the calculated amount of AEDTA was mixed with deionized water and then methyl-β-cyclodextrin was added. For example, Solution No. 1 in Table 2 was prepared by mixing 0.64 mg of AEDTA in 20 mL of deionized water and then adding 65.45 mg of methyl-β-cyclodextrin. The obtained solutions were stirred overnight at room temperature with a stirring rate of 200 rpm. After that, they were lyophilized for two days and then pounded in a mortar, obtaining a homogeneous white powder.

### 3.3. Preparation of the Physical Mixture

The physical mixture was prepared by adding AEDTA and methyl-β-cyclodextrin in a mortar in a weight ratio of 1:10. The two powders were mixed until a homogeneous mixture was obtained.

### 3.4. Formulation of the Coatings

The solutions were prepared by adding the various ingredients in a vial in the following order: acrylic resin, additives, and finally the solvent. They were stirred for 24 h until complete solubilization.

Four types of solutions were prepared containing 0.5%, 1.5%, and 2% *w*/*w* of the methyl-β-cyclodextrin /AEDTA complex (1:1 molar ratio) in a 10% *w*/*w* Paraloid^®^ B44 solution in 1-methoxypropan-2-ol, and, for the fourth solution, 0.5% *w*/*w* of uncomplexed AEDTA in a 10% *w/w* Paraloid^®^ B44 solution.

The solutions were applied by brush on polished bronze discs or poured with the aid of a pipette on glass slides or silicon supports and left to dry until complete solvent evaporation and formation of a thin solid film.

### 3.5. Instrumentation

#### 3.5.1. Fourier-Transform Infrared Spectroscopy (FTIR)

FTIR was used for chemical characterization and to monitor chemical changes over time. All coatings were applied on silicon supports and analyzed in transmission mode with a Perkin Elmer Spectrum 100 spectrometer equipped with a DTS detector (Waltham, MA, USA). The thickness of the coatings was controlled by assuring that the absorbance of the most intense peak was between 0.7 and 1. Each analysis was performed carrying out 16 scans in a range from 450 cm^−1^ to 4500 cm^−1^, with a resolution of 4 cm^−1^. For the ATR-FTIR analyses of the powders, the range was from 650 cm^−1^ to 4500 cm^−1^, with a resolution of 4 cm^−1^. For analyses on polished bronze discs, a Bruker Alpha II spectrometer (Bruker Optics, Entrigen, Germany) operating in external reflection mode was used, collecting 32 scans with a resolution of 4 cm^−1^ in a range from 400 cm^−1^ to 4000 cm^−1^.

#### 3.5.2. X-Ray Diffraction (XRD)

XRD analyses were performed in capillary mode using a Malvern Panalytical X’Pert diffractometer (Malvern, UK) with Cu Kα1 radiation as the source. Measurements were taken across an angular range of 5° to 50° 2θ, with a step size of 0.017° 2θ, and a duration of 79.95 s per step. Baseline correction was applied to each diffractogram using X’Pert HighScore 2.2.1 software.

#### 3.5.3. Nuclear Magnetic Resonance (NMR) Spectroscopy

NMR Spectroscopy was used to investigate the nature of the interaction between the host and the guest and to study the stoichiometry of the complex. The 13 solutions listed in Table 1 were analyzed.

Solution ^1^H NMR spectra were acquired on a Bruker 400 instrument operating at 400 MHz (Bruker Corporation, Billerica, MA, USA) and processed with MestReNova software (v1.19, Santiago de Campostela, Spain). A total of 10 mg of complex was dissolved in 0.6 mL of D_2_O. For pure compounds, the concentration of the solution in D_2_O was kept constant at 3 mM.

#### 3.5.4. Thermal Desorption-Gas Chromatography/Mass Spectrometry (TD-GC/MS)

TD-GC/MS analyses were used to ascertain the presence of the corrosion inhibitor in coatings over time. Sampling for TD-GC/MS analyses was carried out on unaged coatings and after 72 h of thermal aging at 80 °C.

The analyses were performed with a micro-furnace Multi-Shot Pyrolyzer EGA/Py-3030D (Frontier Lab, Koriyama, Japan) coupled to a GC/MS system. Samples were placed into a stainless-steel cup and inserted into the micro-furnace. The desorption temperature was set at 250 °C and kept constant for 1 min. The interface temperature of the pyrolizer was 280 °C and the temperature of the GC injector was kept at 280 °C. The GC was equipped with a methylphenyl-polysiloxane cross-linked 5% phenyl methyl silicone (30 m, 0.25 mm i.d., 0.25 µm film thickness) capillary column. The carrier gas was helium (1.0 mL/min) and the split ratio was one-twentieth of the total flow. The following temperature program was used for the gas chromatographic separation: isotherm of 2 min at 50 °C, ramp of 10 °C/min up to 300 °C, and isotherm at 300 °C for 10 min. An Agilent 8860 Gas Chromatograph and a 5977B Mass Selective Detector (Agilent Technologies, Santa Clara, CA, USA) were used. Mass spectra were recorded under electron impact at 70 eV, with a scan range of 45–800 *m*/*z*. All instruments were controlled by Agilent Mass Hunter Workstation (ver. 10.1.49) software. The mass spectra assignment was done by mass library searches (NIST2008), by comparison with the literature data and the interpretation of fragmentation paths of mass spectra.

#### 3.5.5. SPME-Gas Chromatography/Mass Spectrometry (SPME-GC/MS)

SPME-GC/MS analyses were used to assess the volatilization of corrosion inhibitors over time. Small amounts of coatings were placed in hermetic vials. The vials were placed in an oven at 80 °C and the head space was monitored every 24 h. The sampling of the head space was performed by exposing a SPME fiber for three minutes out of the oven.

The analyses were performed with a 6890 Series Gas Chromatographer system coupled with a 5973 Network Mass Detector (Agilent Technologies, Santa Clara, CA, USA). The capillary column used was the Agilent HP 5MSUI (30 m, 0.25 mm i.d., 0.25 µm film thickness). Supelco SPME fibers in PDMS/DVB (polydimethylsiloxane/divynilbenzene), with a diameter of the fiber 65 µm, were used.

The exposure time of the fiber inside the injector was 30 s. The column temperature was maintained at 50 °C for 4 min and then increased from 50 °C to 250 °C at a rate of 10 °C per minute and maintained at 250 °C for 5 min. All instruments were controlled by Enhanced Chem Station (ver. 9.00.00.38) software.

## 4. Conclusions

A comprehensive study of the methyl-β-cyclodextrin /AEDTA complex was carried out to evaluate its potential as a corrosion inhibitor for bronze. The results demonstrated the formation of an inclusion complex with a 1:1 stoichiometry, in which AEDTA interacted with the internal cavity of the cyclodextrin through the NH_2_ side group. The toroidal structure of the cyclodextrin and its size compatibility with AEDTA facilitated this process. Reasonably, the primary driving force in the formation of the complex was the displacement of high-enthalpy water molecules from the cyclodextrin cavity as the less polar guest molecule entered.

The efficacy of the complex in extending the retention of the corrosion inhibitor within the protective acrylic coatings was evaluated by thermally aging the coatings at 80 °C and monitoring the release of AEDTA in both uncomplexed and complexed forms. These results were also compared to the commercial product Incralac^®^, which contains BTA as a corrosion inhibitor.

The research herein reported demonstrates that methyl-β-cyclodextrin/AEDTA complexes might be promising candidates for the development of novel coatings with enhanced stability, prolonged retention of AEDTA, and other valuable properties such as cost-effectiveness, environmental compatibility, and ease of handling. Further investigation into the anticorrosive properties of these protective coatings is currently underway.

## Figures and Tables

**Table 1:** Chemical shifts (δ) of methyl-β-cyclodextrin and chemical shifts displacements (Δδ) in the complex (1:1 molar ratio).

Methyl-β-Cyclodextrin	H_1_	H_1us_	H_3s_	H_3us_	H_5_, H_6_	H_4_	H_2us_	H_CH3_	H_2s_
δ (ppm)	5.313	5.131	4.636	4.401	3.926	3.712	3.664	3.624	3.445
Δδ _free-complex_ (ppm)	−0.007	−0.002	0.558	0.346	0.006	0.006	−0.006	0	−0.007

**Table 2:** Molar concentrations of the methyl-β-cyclodextrin /AEDTA complex solutions.

Solution No.	AEDTA (mM)	Methyl-β-Cyclodextrin (mM)
1	0.25	2.75
2	0.50	2.50
3	0.75	2.25
4	1.00	2.00
5	1.25	1.75
6	1.50	1.50
7	1.75	1.25
8	2.00	1.00
9	2.25	0.75
10	2.50	0.50
11	2.75	0.25
12	0.00	3.00
13	3.00	0.00

## Data Availability

The raw data supporting the conclusions of this article will be made available by the authors on request.
